# Establishment of an Integrated Model for Predicting Compound Mutagenicity with a Feature Importance Analysis

**DOI:** 10.1021/acs.jcim.5c01586

**Published:** 2025-10-21

**Authors:** Chao-Hsu Yang, Tony Eight Lin, Jui-Hua Hsieh, Kai-Cheng Hsu, Pei-Te Chiueh

**Affiliations:** † Graduate Institute of Environmental Engineering, College of Engineering, 33561 National Taiwan University, 71, Chou-Shan Road, Da’an Dist., Taipei 106, Taiwan; ‡ Graduate Institute of Cancer Biology and Drug Discovery, College of Medical Science and Technology, 535192 Taipei Medical University, No.250, Wuxing St., Xinyi Dist., Taipei 110, Taiwan; § Ph.D. Program for Cancer Molecular Biology and Drug Discovery, College of Medical Science and Technology, Taipei Medical University, No.250, Wuxing St., Xinyi Dist., Taipei 110, Taiwan; ∥ Division of Translational Toxicology, National Institute of Environmental Health Sciences, National Institutes of Health, 111 TW Alexander Drive, Durham, North Carolina 27709, United States; ⊥ TMU Research Center of Cancer Translational Medicine, 38032 Taipei Medical University, No.250, Wuxing St., Xinyi Dist., Taipei 110, Taiwan; # Cancer Center, Wan Fang Hospital, Taipei Medical University, No.111, Sec. 3, Xinglong Rd., Wenshan Dist., Taipei City 116, Taiwan

## Abstract

Assessing the mutagenicity of chemical compounds is crucial for ensuring their safety and minimizing potential environmental and public health risks. However, traditional mutagenicity assessments, such as the Ames test, are time-consuming, resource-intensive, and often limited in their capacity to screen a large number of compounds. To address this gap, predictive models powered by deep learning offer a promising alternative for rapid and cost-effective mutagenicity screening. In this study, we propose an integrated deep learning framework utilizing diverse molecular features to predict compound mutagenicity. In the total usage of 5866 compounds, 5279 compounds were utilized for model training, and the other 587 compounds were utilized for model evaluation. A total of 78 integrated models were developed by systematically combining 13 types of molecular descriptors and fingerprints. The MACCS-Mordred model demonstrated the best performance, achieving a balanced accuracy of 0.885 and a precision score of 0.922 in the testing data set. In addition, we performed an activity cliff analysis to examine potential sources of mispredictions. Applicability domain analysis further confirmed the robustness of the model, indicating that most compounds in our data set fell within the reliable prediction space. Notably, feature importance analysis revealed that mutagenic compounds are more likely to contain nitrogen-containing and ring-related substructures, offering insights into structural characteristics associated with mutagenic risk. Our results support AI-enabled screening tools for prioritizing hazardous compounds and improving early stage chemical risk assessment. This work provides practical value for environmental monitoring and regulatory decision-making.

## Graphical abstract

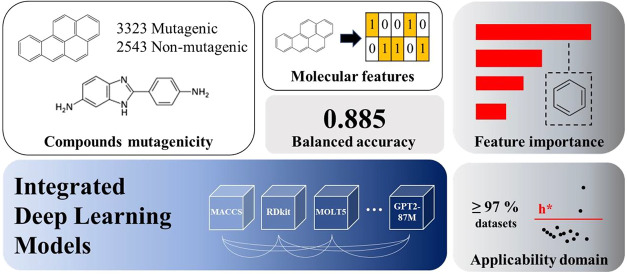

## 1 Introduction

Mutagenicity is a concerning hazardous end point that presents the capacity of a compound to induce mutations in deoxyribonucleic acid (DNA) sequences.^1–3^ Compounds inducing mutagenicity have the potential to pose long-term risks to living beings, causing heritable mutations in germ cells and cancer in somatic cells.^4^ Chemical-related organizations and policies worldwide have regulated the assessment of mutagenicity as an essential requirement for the safety of chemical compounds, drug candidates, and consumer products.^5,6^ Among all detection methods, the Ames test is considered the standard assay for mutagenicity.^7–9^ The Ames test adopts at least five different cell strains for mutagenicity evaluation, of which four should be the assigned strains (TA1535, TA1537 (or TA97a or TA97), TA98, and TA100). If at least one result is positive in all tested cell strains, a compound would be considered a mutagen. These requirements have significantly enhanced the reliability and reproducibility of the Ames test, making it a widely utilized method for regulatory purposes before registering new and existing compounds.

The accumulated cost and time involved in the Ames assessment are becoming crucial as approximately 4000 emerging compounds are added to the registry daily.^10^ Therefore, this has led to a growing interest in *in silico* methods due to their rapid speed and cost-effectiveness. The most applied computational method for mutagenicity prediction is the quantitative structure–activity relationship (QSAR) model. In a QSAR model, the molecular descriptors are used to represent the characteristics of chemicals,^11,12^ and computational approaches such as machine learning (ML) algorithms are applied to calculate the sophisticated quantitative relationship between mutagenicity and molecular descriptors.^10^ For instance, the compound mutagenicity can be predicted by the presence of expert-rule-based substructures (*i.e.*, structural alerts) and certain types of molecular fragments,^13^ or via the quantified statistical correlations between molecular descriptors and mutagenicity.^14^ In addition, a vast number of molecular descriptors have been explored in recent years,^15^ various commercial QSAR models (*e.g.*, CASE ultra and VEGA) have been established and proven their effectiveness.^16–18^ Furthermore, an increasing use of machine learning and deep learning in cheminformatic modeling can be observed in the past decades.^19,20^ These studies showed that the current development of QSAR and chemoinformatic models has demonstrated the possibility of mitigating the required costs involved in assessment due to their efficient and accurate predictive abilities.

Deep neural networks (DNNs) have become a common approach in various predictions due to their excellent ability to process large and complex data sets. Specifically, they excel in analyzing features and their connections to mutagenicity.^19,21,22^ For example, in a comparative study involving 4053 compounds, deep learning models outperformed traditional machine learning algorithms in predicting mutagenicity.^4^ Similarly, the application of a message passing neural network, which represents an advanced form of graph neural networks, demonstrated superior performance in predicting mutagenicity as well as six other types of toxicity.^23^ However, most models were generally constructed solely on one specific type of molecular features.^24^ The integrated modeling approaches—which combine the outputs of multiple models built on diverse feature types—have been suggested to improve predictive performance^25,26^ Building on this rationale, an integrated model has the potential to achieve superior accuracy in mutagenicity prediction.

In this study, we aimed to establish an integrated DNN model to predict mutagenicity (Figure 1). Compounds from three databases were collected and split into training and testing data sets to establish and evaluate the models. Various models were established following the engineering of molecular features. Each model was optimized by tuning the hyperparameters. Subsequently, these optimized models were combined pairwise to integrate their predictions, and the best-integrated model was selected depending on the score metrics at cross-validation. An analysis of feature importance was conducted afterward to unveil the relationship between molecular descriptors and mutagenicity. In addition, the applicability domain (AD) was implemented to identify the reliable prediction region of the constructed model. We believe that the integrated model will be useful in reducing costs and accelerating the mutagenicity assessments.

**Figure 1:**
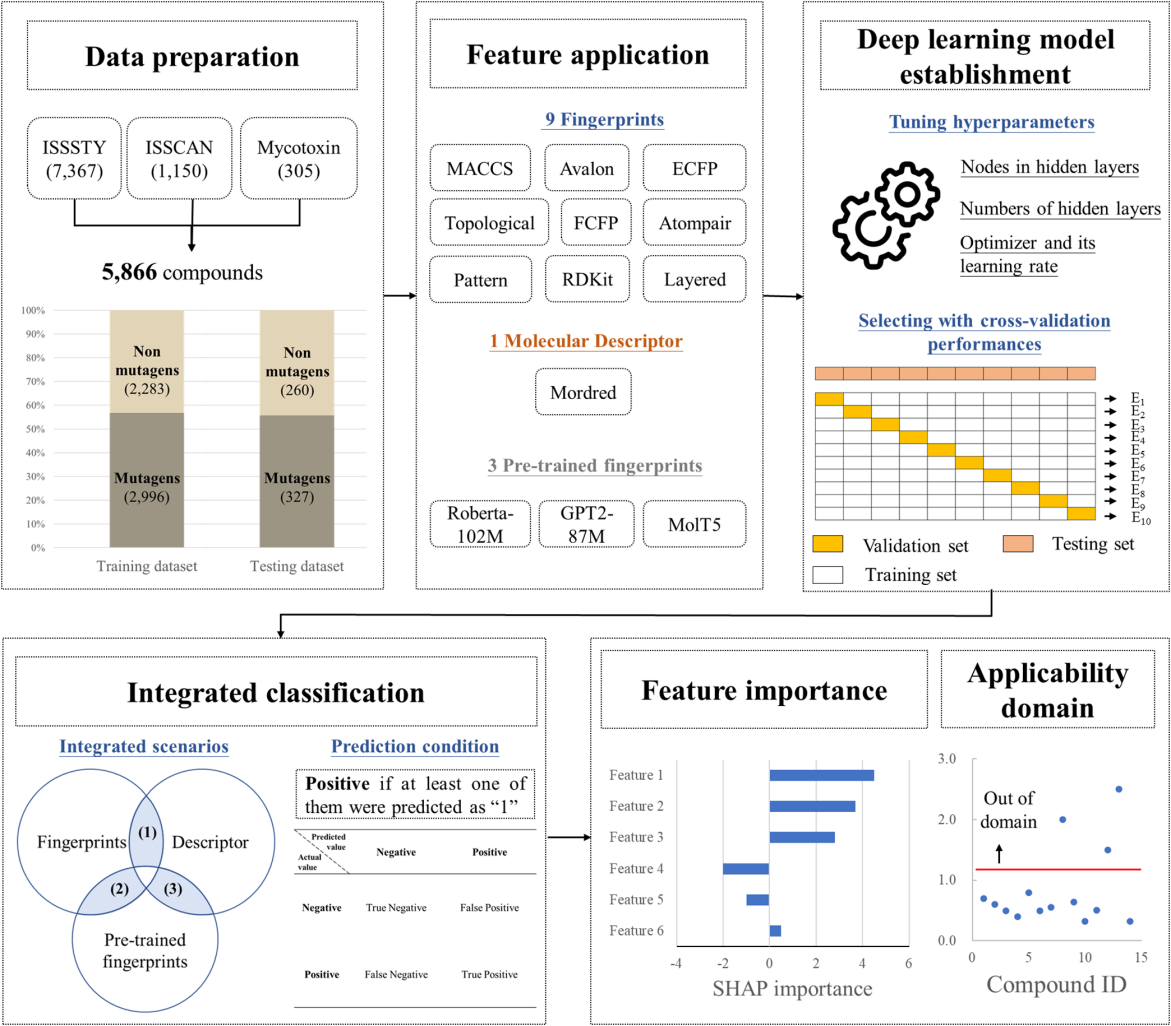
Workflow of the study. Compounds with mutagenicity data were collected from public libraries. Molecular features were generated and used to establish deep-learning models. Models were optimized by tuning hyperparameters through cross-validation. Optimized models were combined in a pairwise manner to generate a integrated model. The best integrated model was then used to analyze feature importance and calculate the applicability domain, ensuring reliable predictions of mutagenicity.

## 2 Material and Methods

### 2.1 Data Collection and Preparation

Compound data sets were gathered from the ISSSTY,^27^ ISSCAN,^27^ and MicotoXilico data sets.^28^ The former two data sets were provided by Istituto Superiore di Sanita’ under the ISSTOX project, and MicotoXilico was compiled from the work of Aydın and Rencüzoğulları^29^ and open-source databases, such as CPDB, CCRIS, and OpenFoodTox. Duplicate compounds were first removed. Next, equivocal labels, as defined by the ISSSTY and ISSCAN data sets (*e.g.*, dimethoate and tribromoacetic acid), were excluded from this study for binary classification. The largest components were retained, and compound structures were standardized and sanitized using the datamol toolkit.^30^ In total, 5866 compounds were included in our data set, with the training and testing data points randomly selected at a 90:10 ratio. The training data set included 5279 compounds, containing 2996 mutagens and 2283 nonmutagens. The testing data set included 587 compounds, containing 327 mutagens and 260 nonmutagens. The principal component analysis (PCA)^31,32^ was utilized for chemical space visualization, where the number of dimensions/principal components was set to two for the PCA plot, and the percentage of retained information was calculated.

### 2.2 Feature Engineering

The molecular descriptors were applied to convert chemical structures into a compatible data format for the model. The details of these features and the number of features used for each model are summarized in Table S1. The fingerprints, including MACCS, Avalon, ECFP, FCFP, AtomPair, Topological, RDKit, Layered, and Pattern were adopted due to their common application in drug discovery and biodegradability prediction models.^33^ The Mordred descriptors were selected for a variety of combinations of constitutional features,^34^ and pretrained fingerprints (*e.g.*, Roberta-Zinc480M-102M, GPT2-Zinc480M-87M, and MOLT5) were selected for their novel generation approach along with language models.^35–37^ In total, 13 different types of descriptors were utilized. All features were generated using Molfeat,^38^ and the missing values were removed.

### 2.3 DNN Model Construction

DNN models were established with Keras API.^39^ Each DNN model could be expressed as a fully connected network where the computing capability depends on various hyperparameters. To optimize the model, the following parameters were arranged: the number of hidden layers ∈ {1, 2, 3}, number of neurons per each hidden layer ∈ {(512,*), (512, 128), (512, 128, 8)}, and the optimizer ∈ {Adamax, Adam}. Other hyperparameters, such as the learning rate and the application of batch normalization, were fixed in this section due to their negligible effects on the learning process during preliminary experiments. In addition, the activation functions for the hidden and output layers were set as ReLU and sigmoid, respectively.

### 2.4 Integrated Model Construction

Integrated models were established by pairwise combining individual DNN models, which was similar to the assessment standard of OECD TG471 guidelines. A compound was labeled as positive (1) if at least one prediction was positive (mutagenic), and a compound was labeled as negative (0) if none of the models predicted it as negative (nonmutagenic). The integrated model that exhibited the best performance was evaluated using the testing data set and utilized for further analysis of feature importance. Six score metrics were used to evaluate the model performances: accuracy, balanced accuracy, precision, recall, F1 score, and the MCC (Matthews correlation coefficient). These metrics were calculated by the number of true positives (TPs), false positives (FPs), true negatives (TNs), and false negatives (FNs). The formulas for these metrics are listed below (eqs 1–6a). Balanced accuracy and precision scores were calculated.

1 $$\mathrm{accuracy}=\frac{\mathrm{TP}+\mathrm{TN}}{\mathrm{TP}+\mathrm{TN}+\mathrm{FP}+\mathrm{FN}}$$

2 $$b\mathrm{alanced accuracy}=\frac{1}{2}\left(\frac{\mathrm{TP}}{\mathrm{TP}+\mathrm{FN}}+\frac{\mathrm{TN}}{\mathrm{TN}+\mathrm{FP}}\right)$$

3 $$\mathrm{precision}=\frac{\mathrm{TP}}{\mathrm{TP}+\mathrm{FP}}$$

4 $$r\mathrm{ecall}=\frac{\mathrm{TP}}{\mathrm{TP}+\mathrm{FN}}$$

5 $$F1=2\times \frac{\mathrm{precision}\times \mathrm{recall}}{\mathrm{precision}+\mathrm{recall}}$$

$$\mathrm{MCC}=\frac{\mathrm{TP}\times \mathrm{TN}-\mathrm{FP}\times \mathrm{FN}}{\sqrt{\left(\mathrm{TP}+\mathrm{FP}\right)\times (\mathrm{TP}+\mathrm{FN})\times (\mathrm{TN}+\mathrm{FP})\times (\mathrm{TN}+\mathrm{FN})}}$$ 6

### 2.5 Feature Importance

The importance and positive/negative contribution of each feature for mutagenicity predictions will be derived from the analysis of the integrated model with the best cross-validation performances. The feature importance was analyzed using the SHAP (SHapley Additive exPlanations) method,^40^ an explainable artificial intelligence technique derived from the cooperative game theory. The SHAP method expresses feature importance as a Shapley value. A feature with a higher absolute Shapley value indicates a greater significance in influencing mutagenicity. Furthermore, a positive Shapley value can be interpreted as a feature that positively influences the final prediction; conversely, a negative Shapley value can be considered a negative contributing factor. In addition, the exploration of how the modification of the identified feature importance affects the compound mutagenicity will be discussed by utilizing the Exmol package,^41^ a technique that generates analogs with different mutagenicity to the given chemical based on Tanimoto similarity and a specified condition equation. The equation is stated below

7 $$m\mathrm{inimize}\;d\left(x,x^{\prime }\right)\;\mathrm{such that}\;f\left(x\right)\neq f\left(x^{\prime }\right)$$

where *x* and *x*′ present the molecular descriptor vectors, *d*(*x*, *x*′) is a measure of distance between the molecular descriptors, and *f*(*x*) and f­(*x*′) are the mutagenicity predictions for the compounds.

### 2.6 Applicability Domain

The prediction reliability of compounds was assessed by the AD, a theoretical chemical space determined by training sets. Reliable predictions are only generated within the AD; predictions outside the AD are considered unreliable.^42^ In this study, the pyADA package,^43^ an open-source Python toolkit, was utilized to determine the AD. The package employed the leverage approach^44^ to calculate the boundary, which is the critical hat value (h*), of the AD and the *h* value for each compound. The h* value is calculated as 3­(*p* + 1)/*n*, where *p* is the feature number in the model and n is the compound number in the training sets.^44^ Compounds with a higher *h* value than h* represent a great structural difference compared to the training sets and are thus considered outside the AD.

## 3 Results

### 3.1 Data Set Analysis

The training and testing sets were obtained independently through random splitting. We first performed a PCA to visualize the chemical space of the compounds. Three of the 13 molecular descriptors, the extended connectivity fingerprint (ECFP), MACCS, and Mordred features of the compounds, were utilized to construct the PCA, mapping the chemical distribution. The PCA results indicated a similar chemical distribution between the training (blue dots) and testing (orange dots) data sets (Figure 2). The chemical space represented by MACCS appeared more sparsely distributed compared to those based on ECFP and Mordred features. All testing data sets derived from these three feature types fell within the domain of their corresponding training data sets. In terms of explained variance, the first two principal components accounted for 1.59% for ECFP, 17.87% for MACCS, and 40.27% for Mordred descriptors. Furthermore, the PCA plots of mutagens and nonmutagens exhibited substantial overlap, suggesting that a nonlinear classification approach may be required to accurately distinguish between the two groups. This similarity supported the robustness of the data set division and indicated a well-balanced representation for model training and evaluation.

**Figure 2:**
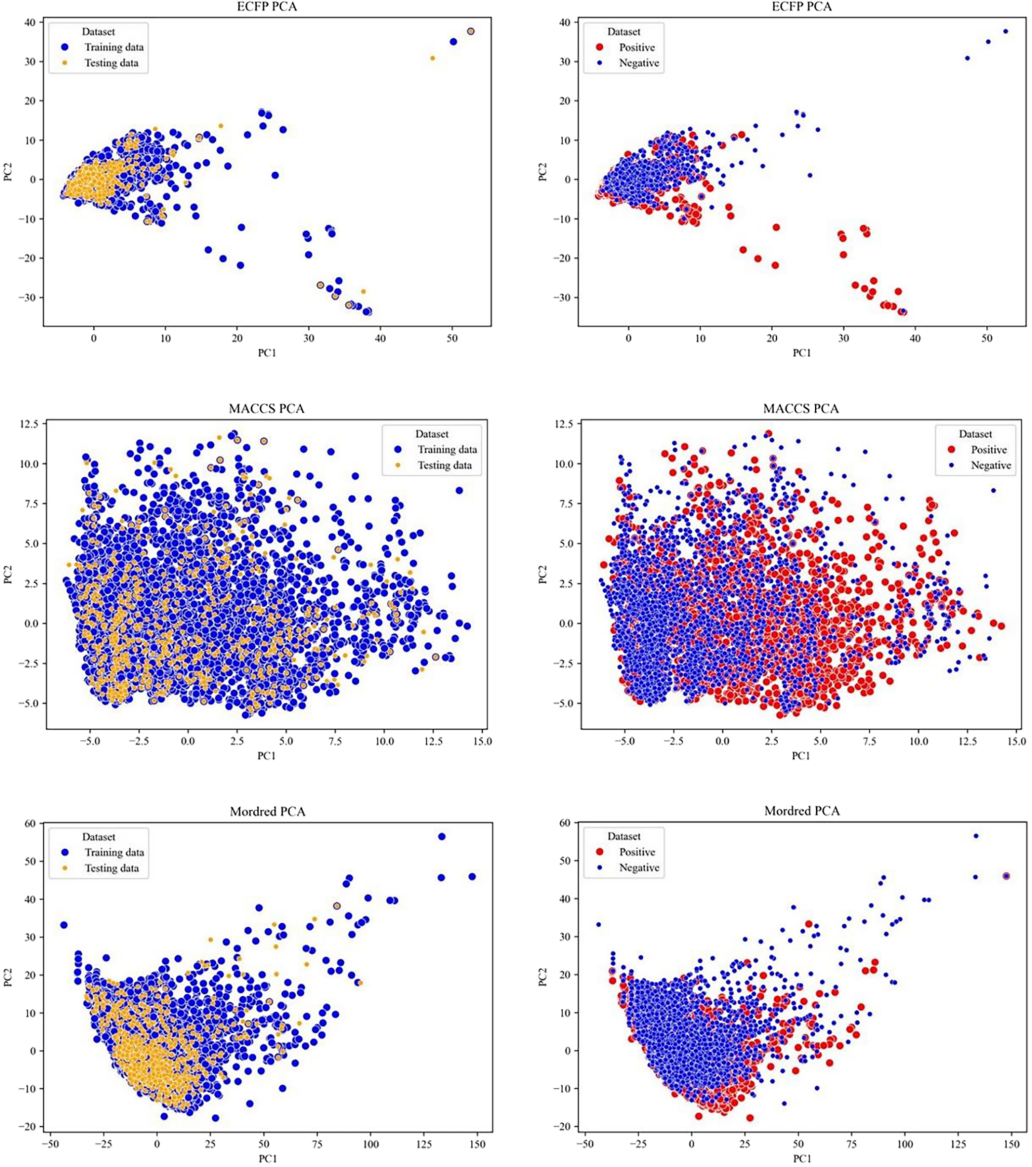
Chemical distribution of the data set presented by the PCA plot. Training and testing sets are respectively presented in blue and orange dots. Mutagens and nonmutagens are respectively presented in red and blue dots.

### 3.2 Model Establishment

Next, after using 13 types of molecular features representing various structural and property aspects of the compounds, we utilized the training set and DNN approach to establish our models. In addition, we tested different network architectures by varying combinations of hidden layers and optimizers. In total, 78 models were established, and their performances were evaluated through 10-fold cross-validation. The results showed that models with three hidden layers exhibited better precision, ranging from 0.840 to 0.890 (Figure 3). In addition, the models using the Adamax optimizer outperformed those with the Adam optimizer, achieving an average accuracy of 0.827, a balanced accuracy of 0.830, a precision of 0.876, a recall of 0.812, and an F1 score of 0.842 (Table 1). As a result, models with three hidden layers and the Adamax optimizer were selected for further study.

**Figure 3:**
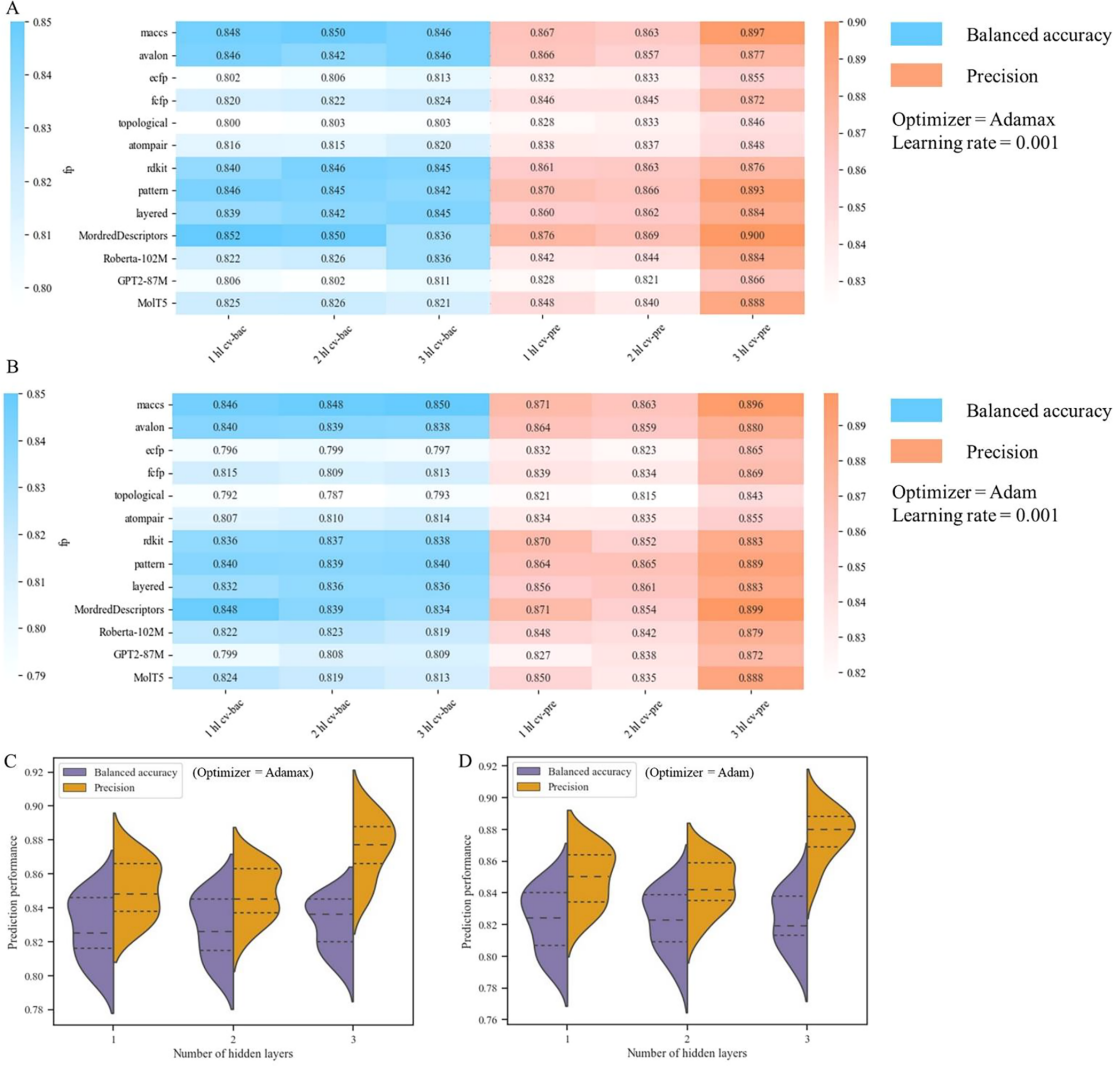
Performance comparison of models generated by various features and hyperparameters. (A, B) The 10-fold cross-validation results of each model between the usage of Adamax and Adam optimizer, and the number of hidden layers utilized by each model. The balanced accuracy was labeled as “X hl cv-bac”, where precision was labeled “X hl cv-pre”. The blue-colored values represent the cross-validation balanced accuracy, and the orange-colored values represent the cross-validation precision. (C–D) The performance violin plots of each model between the usage of Adamax and Adam optimizer.

**Table 1:** Model Performance Obtained by Averaging Results of 10-Fold Cross-Validation

optimizer	accuracy	balanced accuracy	precision	recall	F1
Adam	0.818 ± 0.018	0.823 ± 0.017	0.877 ± 0.015	0.792 ± 0.026	0.832 ± 0.018
Adamax	0.827 ± 0.015	0.830 ± 0.015	0.876 ± 0.017	0.812 ± 0.024	0.842 ± 0.014

We further compared the performance of models established using various molecular features. The models utilizing substructure fingerprints (*e.g.*, MACCS, RDKit, Layered, and Pattern) and the Mordred descriptor generally outperformed those based on circular fingerprints (*e.g.*, ECFP and FCFP) and path-based fingerprints (*e.g.*, Topological and Atompair) (Figure 3). Among the models, those using the MACCS fingerprint and Mordred descriptor exhibited the best performance. The MACCS fingerprint model achieved the highest balanced accuracy of 0.846 and precision of 0.900, while the Mordred descriptor model showed balanced accuracy of 0.836 and precision of 0.900. A previous study has noted that random data set splitting can sometimes result in higher error variance if the resulting subsets fail to capture the diversity of the original data set, particularly when the data are complex or unevenly distributed.^45^ Nevertheless, our findings indicate that models trained on randomly split data sets in this study achieved promising predictive performance. In addition, our models had low standard deviations during the cross-validation. Taken together, the comprehensive analysis of various molecular feature-based models suggested that the MACCS fingerprint and Mordred descriptor models were reliable and had the potential for predictive applications.

### 3.3 Integrated Model Performances

We combined the 13 individual models in a pairwise manner to develop integrated models due to the enhanced performance potential of integrated models that merge different prediction models. In the integrated models, a compound was classified as positive for mutagenicity if any model identified the compound as mutagenic and classified it as negative if all models concluded the compound was nonmutagenic. This classification aligned with the standards specified in OECD TG471 guidelines. A total of 78 integrated models were established, and their performances were evaluated using a 10-fold cross-validation method (Figure 4). The results showed that integrated models generally exhibited better balanced accuracy than one-feature models (Figure 4). Several models demonstrated high accuracy and precision, exceeding 0.84. These models generally utilized a combination of different types, including substructure fingerprints and the Mordred descriptor. Among all established integrated models, we utilized a multicriteria decision-making strategy to select the best model. By considering the cross-validation accuracy, balanced accuracy, and precision of each integrated model, the average of these metrics was used for the model decision. As shown in Table S2, the MACCS-Mordred model had an average performance of 0.857, superior to the other top 10 integrated models. This suggests that integrating various predictive models can enhance their predictive capabilities, and shows that the MACCS-Mordred model has the potential for further application.

**Figure 4:**
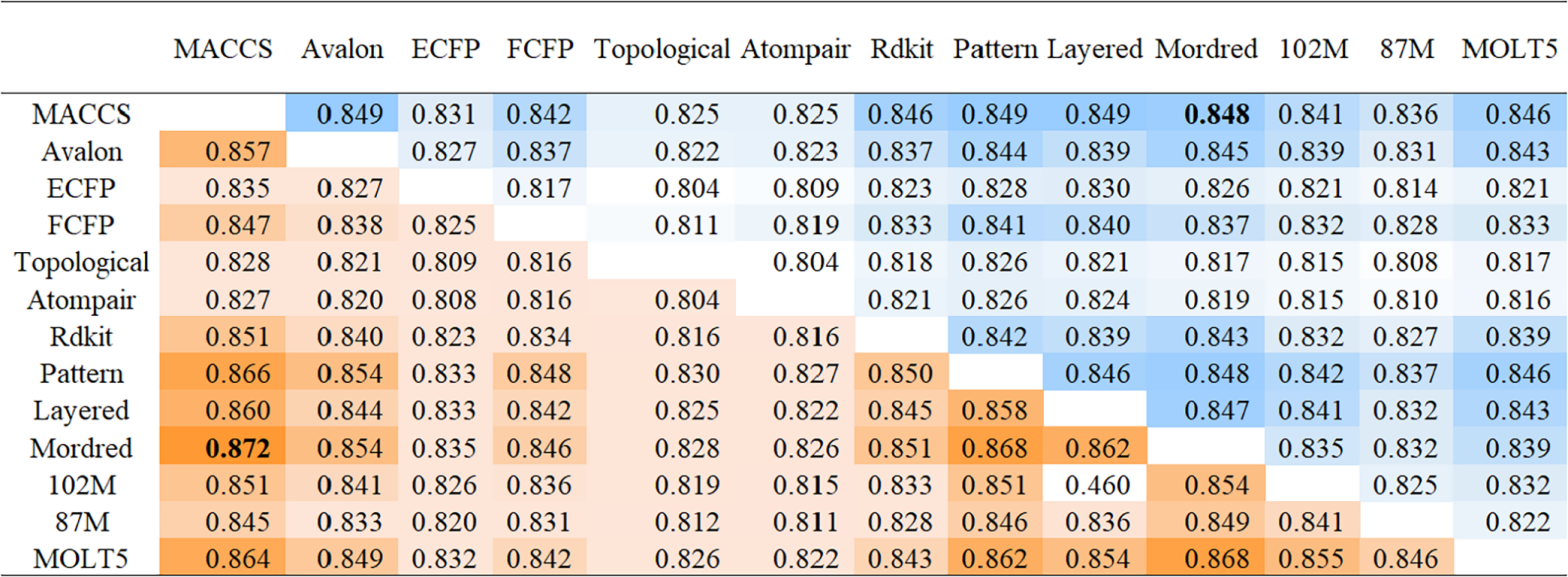
Performance of integrated models combining pairwise models. The performance results are from 10-fold cross-validation. The upper right section (cells colored in blue) presents the balanced accuracy of the integrated models, and the bottom left section (cells colored in orange) shows the precision of the integrated models.

The results for the integrated MACCS and Mordred model and the individual MACCS and Mordred models are shown in Table 2, and the results for other single-feature models are shown in Table S3. For the training set, the integrated model achieved scores of 0.926 for accuracy, 0.929 for balanced accuracy, 0.963 for precision, 0.904 for recall, and 0.932 for F1 score. The models were then evaluated using the testing set. Consistent with the results of the training set, the integrated model had better performance compared to the individual models, achieving an accuracy of 0.882, a balanced accuracy of 0.885, a precision of 0.922, a recall of 0.862, and an F1 score of 0.891. The results suggested that the integrated model was reliable for predicting mutagenicity.

**Table 2:** Performance of the Selected Integrated Model^a^

	training set						testing set					
	Acc	Bal acc	precision	recall	F1	MCC	Acc	Bal acc	precision	recall	F1	MCC
MACCS model	0.911	0.918	0.974	0.866	0.917	0.828	0.872	0.879	0.944	0.820	0.877	0.753
mordred model	0.896	0.905	0.976	0.837	0.902	0.804	0.850	0.860	0.944	0.777	0.852	0.717
integrated model	0.926	0.929	0.963	0.904	0.932	0.852	0.882	0.885	0.922	0.862	0.891	0.766

a The “Acc” represent the accuracy, “Bal acc” represent the balanced accuracy.

### 3.4 Individual Compound Mutagenicity Impacts of Chemical Descriptors

Feature importance was analyzed using the SHAP method to identify critical features affecting the potential for mutagenicity of a compound. The features included MACCS fingerprints and Mordred descriptors. A feature was considered to have a greater impact on mutagenicity if it exhibited a high SHAP value. The top 20 key positively contributing features of MACCS fingerprints ranked by their SHAP values are shown in Figure 5A and can be categorized into four groups according to their structural characteristics: (1) halogen group, (2) nitrogen-containing group, (3) oxygen-containing group, and (4) ring-related group (Figure 5B). First, the halogen group consisted of features including halogen atoms of fluorine (F), chlorine (Cl), bromine (Br), and iodine (I). For example, the fluorine atom (*i.e.*, MACCS134) of the compound *N*-(4-fluorophenyl)-5-nitro-3-thiophenecarboxamide was identified as a key feature inducing mutagenicity (Figure 5C). The nitrogen-containing group included eight features, such as NH_2_(MACCS84), A$A!N­(MACCS133) and N=O­(MACCS63). These features also had a notably high incidence of mutagenicity. For example, 86.2% of compounds containing the N=O­(MACCS63) feature were mutagens, as observed in the compound 4-amino-3-nitro-6-chloroaniline (Figure 5C). The oxygen-containing group included four features: QO (MACCS102), O=A > 1 (MACCS136), OAAO (MACCS72), and A!O!A (MACCS126), which are branched functional groups. For instance, Menogaril contained two of the features, viz., OAAO (MACCS72) and A!O!A (MACCS126), both of which are connected to the main scaffold of the molecule. The last ring-related group comprised six features, such as the 3 M ring (MACCS22) and the aromatic ring (MACCS125). For example, the compound anti-5,7-dimethylchrysene-1,2-diol-3,4-epoxide simultaneously contained multiple features (Figure 5C) of this group, contributing to its mutagenicity. The analysis highlighted the importance of the molecular features in understanding chemical mutagenicity.

**Figure 5:**
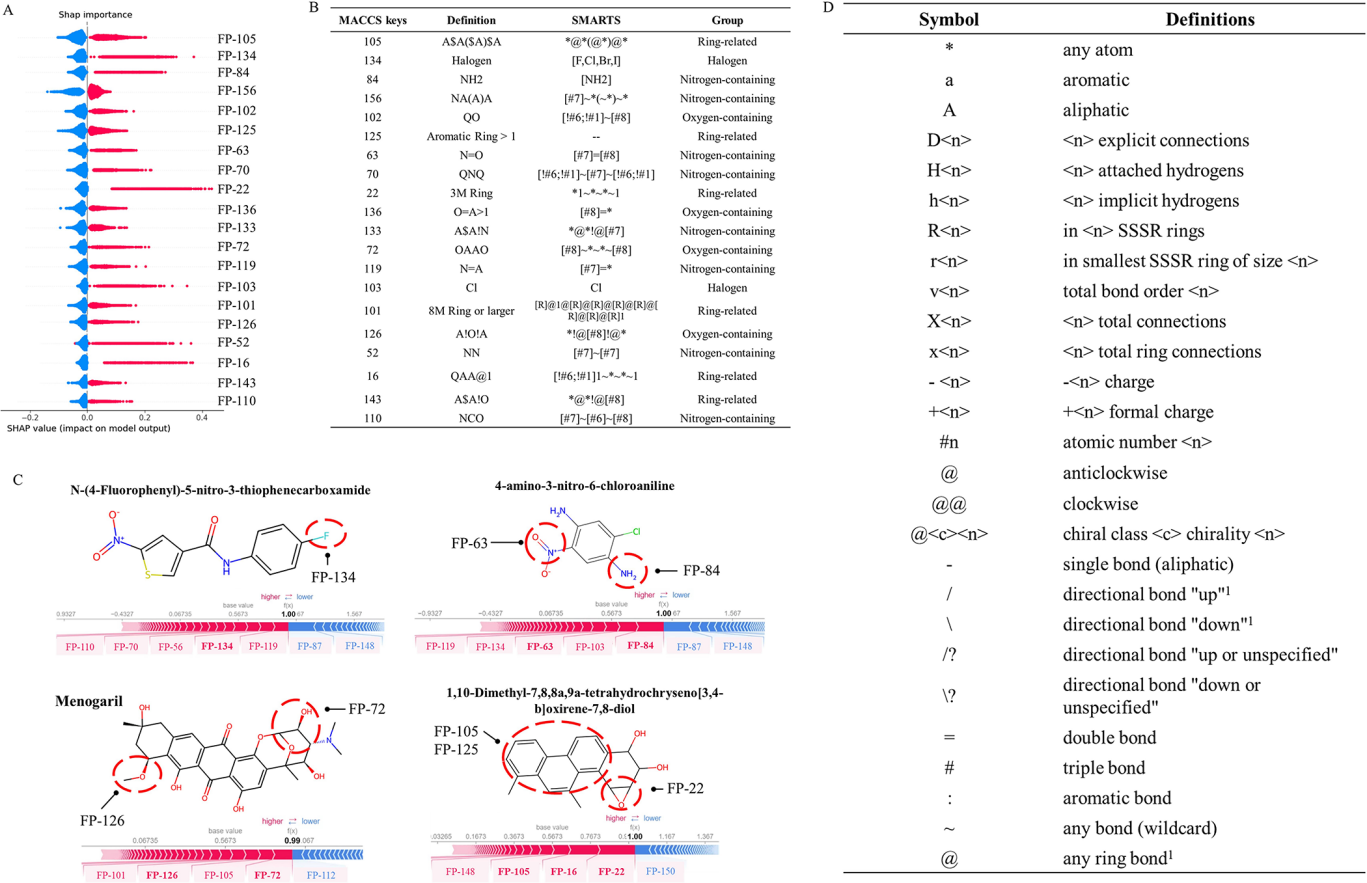
Feature importance analysis for MACCS fingerprint. (A) Top 20 important features showing a positive contribution to mutagenicity. (B) MACCS keys, definition, SMARTS presentations, and groups of features. (C) Examples of mutagenic compounds. The red dashed circle indicates features inducing mutagenicity. Red text represents features with a positive contribution, while the blue text indicates negative contributions. (D) Definitions of the SMARTS pattern.

Next, we analyzed the importance of Mordred descriptors, and the top 20 positively contributing features ranked by SHAP values were identified (Figure 6A). These features were categorized into four groups using a classification approach similar to the method used for grouping MACCS fingerprints: (1) ring-related group, (2) nitrogen-containing group, (3) oxygen-containing group, and (4) others (Figure 6B). The ring-related group included five features, such as the total valence electrons (*i.e.*, E-state values) for “aaCa” groups in compounds (SaaaC) and the number of 12-or-greater-membered fused rings (nG12FRing). For example, the compound 7-ethylbenz­[*a*]­anthracene 5,6-imine included multiple features from this group, contributing to its mutagenicity (Figure 6C). The nitrogen-containing group included two features: the sum of valence electrons in NH2 (SsNH2) and the number of Y-shaped connected nitrogen atom (NsssN). In the compound 2-(butylnitroamino)­ethyl nitrate, the presence of nitrogen atom connected to the nitrite functional group was identified as a key factor in inducing mutagenicity (Figure 6C). The sum of valence electrons in the “–O–” group (SssO) was the only member in the oxygen-containing group, and the compound fenitrothion contained that, which contributed to its mutagenic properties (Figure 6C). The last group included molecular features, such as electrical state indices and van der Waals surface area contribution (VSA_EState4), log *P* and surface area contribution (*S* Log *P*_VSA8), and topological charges (JGI8). For example, the compound 2-iodo-9-acridinamine had a high VSA_Estate 4 value. This analysis identified key Mordred descriptors, thereby enhancing our understanding of molecular features crucial for predicting mutagenicity.

**Figure 6:**
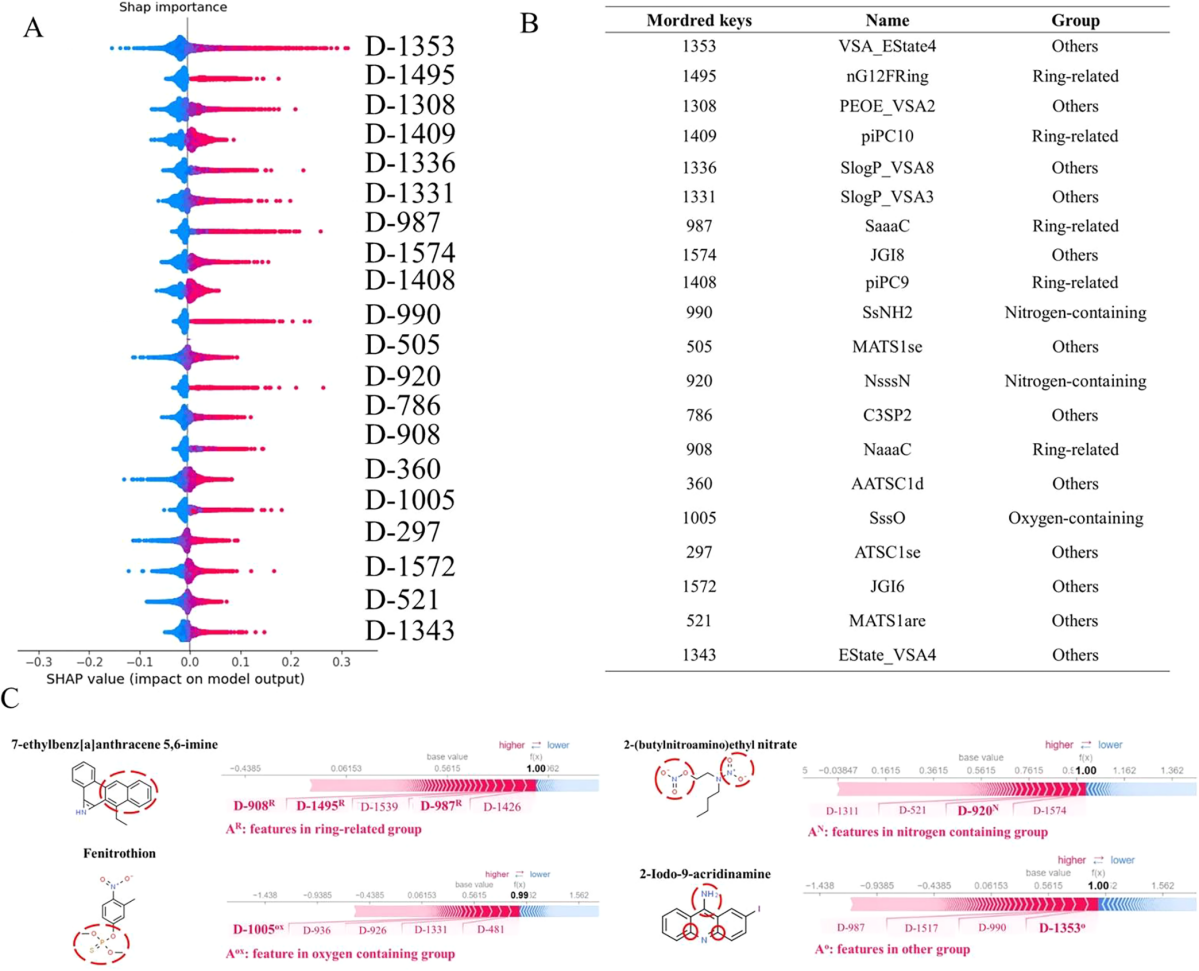
Feature importance analysis for Mordred descriptors. (A) Top 20 important features showing a positive contribution to mutagenicity. (B) Mordred keys, names, and groups of features. (C) Examples of mutagenic compounds. The red dashed circle indicates features inducing mutagenicity. Red and blue text respectively indicate features with positive and negative contributions.

### 3.5 Model Applicability Analysis

The MACCS and Mordred features applied in the final model were utilized to analyze the AD, which demonstrates the confidence region for predicting new compounds. A compound was considered to be within the AD if its hat value (*h*) was below the threshold (h*) using the leverage method. The pyADA package was utilized to calculate the AD.^43^ The results showed that most compounds were located in the AD when analyzed using MACCS and Mordred features. For MACCS features, 127 compounds (*i.e.*, 2.165% of the data set) had hat values that exceeded the threshold (h*) of 0.095. Meanwhile, only eight compounds were found outside the AD when applying a threshold of 0.6 (Figure 7). The AD details of other established models were summarized in Table S4. Furthermore, these “outliers” detected by the ADs were not the same, suggesting that our integrated model has the potential to correct these mispredictions (Figure S1A). For instance, among the MACCS-AD-identified outliers, three of the compounds had a misprediction by the MACCS model, but they were correctly predicted in our final integrated model since they were within the AD derived from Mordred descriptors (Figure S1B). In addition, all outliers identified by the Mordred-defined AD were correctly predicted by our final model, suggesting that their mutagenicity evaluation in the MACCS model was reliable due to their presence within the AD (Figure S1C). The results suggested that the data set exhibited a high overlap in chemical space distribution, and new compounds within the AD could be predicted with high reliability.

**Figure 7:**
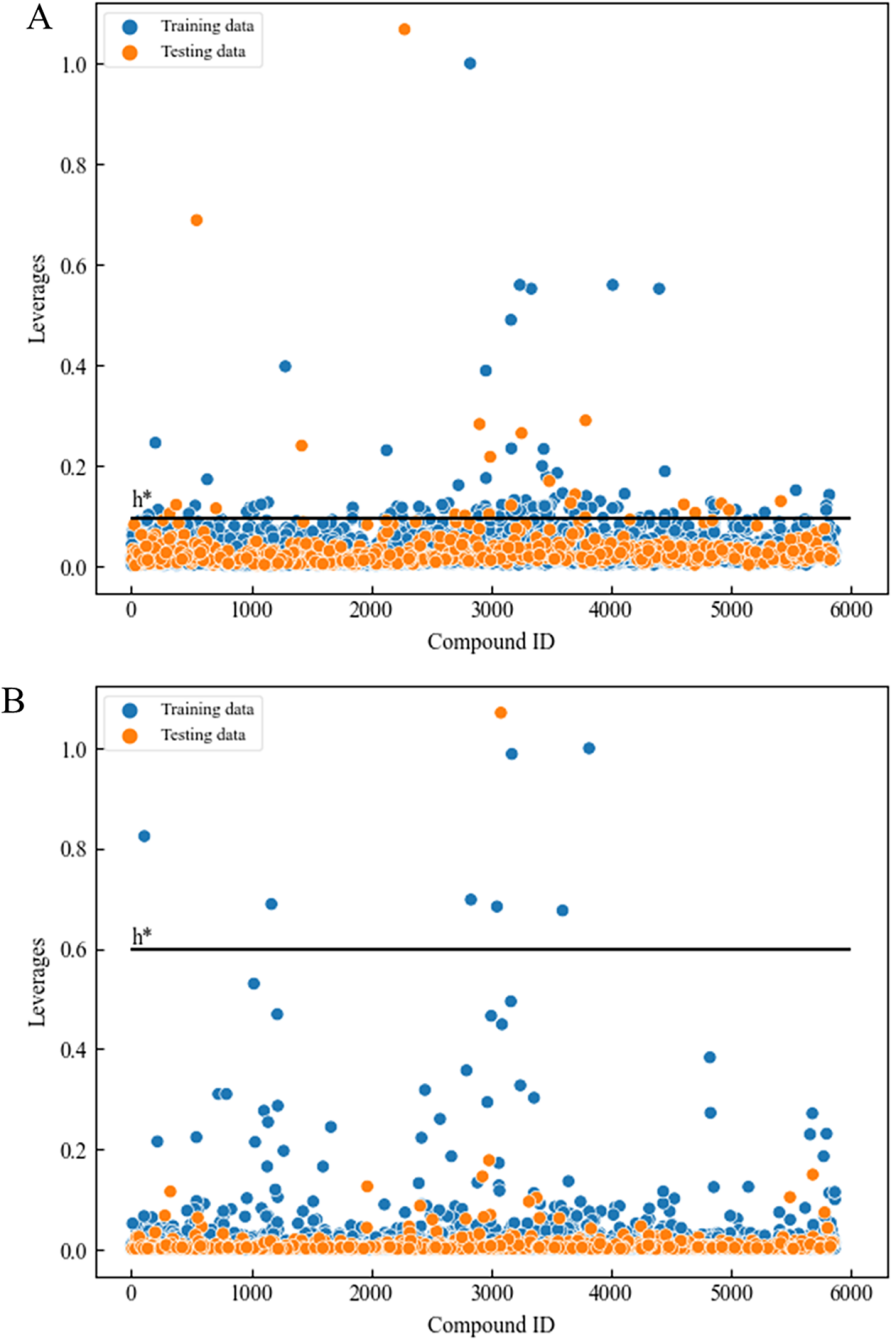
Analysis of the applicability domain. (A) Utilizing the MACCS fingerprints, the applicability domain boundary (h*) was calculated to be 0.095. (B) Utilizing the Mordred descriptors, the applicability domain boundary (h*) was calculated to be 0.6.

### 3.6 Model Application of Chemicals beyond Our Data Set

To test the model application in case studies not present in our data set, we selected chloranil,^46^ 5-nitro-2-propoxyaniline,^47^ and syn-dibenz­[*a*,*j*]­acridine-3,4-diol-1,2-epoxide^48^ for evaluating their mutagenicity. The results showed that the three chemicals were predicted to be mutagenic, consistent with their experimental mutagenicity. Furthermore, the chemicals were located within the AD defined by the MACCS and Mordred descriptors (Table S5). These findings demonstrated our integrated model could be applied to known mutagenic compounds beyond our data set, and the mutagenicity predictions from our integrated model were reliable while the compounds were within the AD.

## 4 Discussion

### 4.1 Comparison of Integrated Model and One-Feature Models

The analysis indicated that the integrated models performed better than the models utilizing a single type of molecular feature. The superior results included accuracy, balanced accuracy, recall, and F1-score. This suggested that using a voting approach similar to the integrated mechanism used in the Ames test, enhanced the accuracy of mutagenicity predictions. The improved performance of the integrated model may be due to the integration of diverse compound features, which provided complementary information for prediction. For instance, the MACCS and Mordred models generated 470 and 532 false predictions in the training set. When the two models were combined, the integrated model resulted in only 392 false predictions. Consistent with training results, in predicting the testing set, the integrated model had only 69 false predictions, whereas the MACCS and Mordred models respectively had 75 and 88 false predictions (Figure 8). Our data set comprised 3323 mutagens and 2543 nonmutagens, which is slightly unbalanced. An unbalanced data set may yield high accuracy but low balanced accuracy. For example, Martinez et al.^1^ reported a mutagenicity prediction accuracy of 0.95 but a balanced accuracy of only 0.71, which was due to the presence of 3103 mutagens among 3334 compounds. Similarly, Li et al.^49^ reported a balanced accuracy of 0.69 when their data set contained 1480 mutagens and 8546 nonmutagens. In contrast, Pandey et al.^3^ reported an accuracy of 0.76. Based on their reported results and data set composition (3503 mutagens and 3009 nonmutagens), the corresponding balanced accuracy can reasonably be considered similar. In our study, the difference between the accuracy and balanced accuracy of the integrated model was only 0.003, indicating that the data set was only mildly unbalanced. These findings not only demonstrated the effectiveness of constructing an integrated model but also proved the complementary functionality with the employment of another feature.

**Figure 8:**
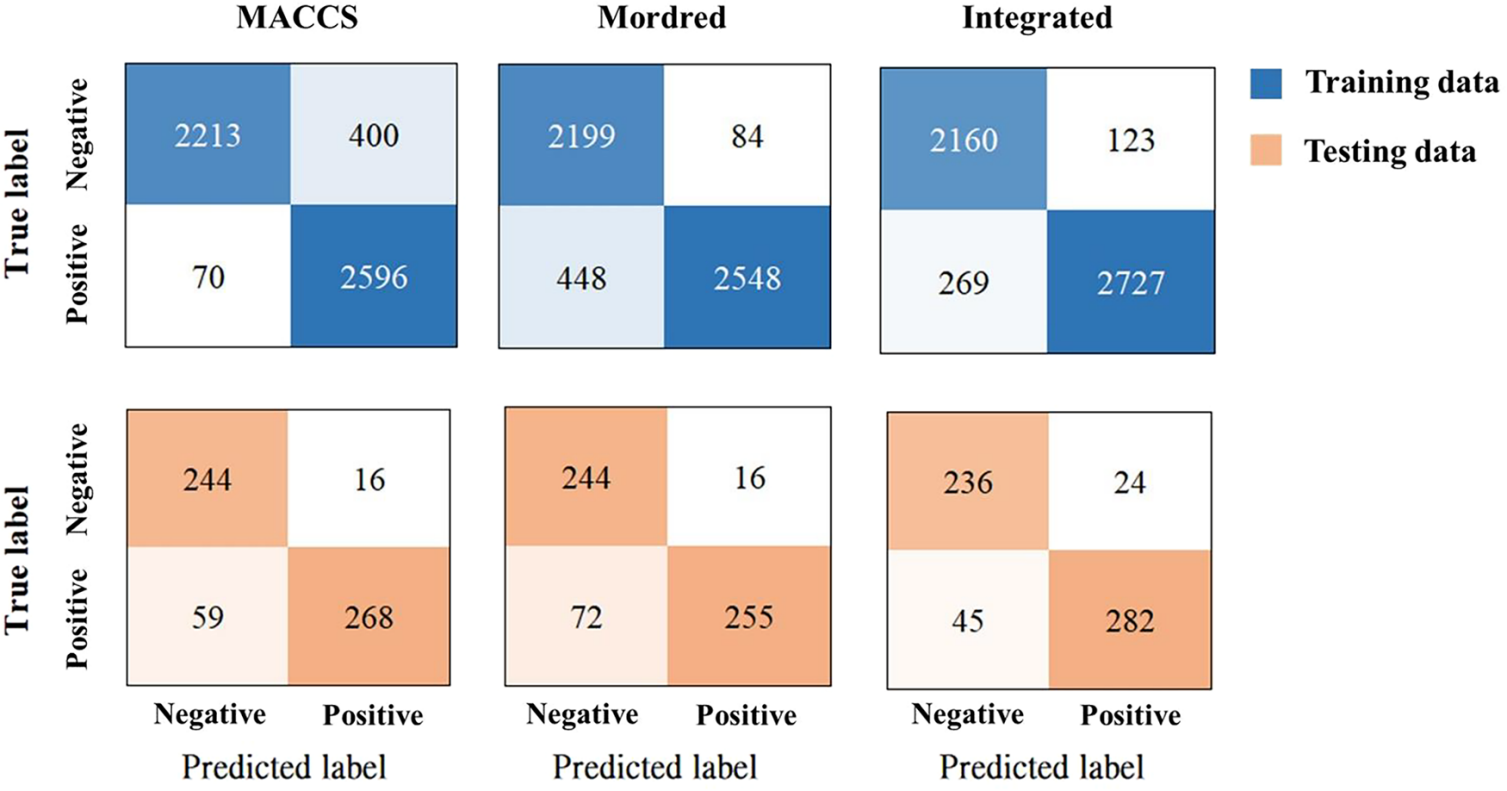
Confusion matrices of the constructed models. The predictions of the training and testing sets are presented in blue and orange cells, respectively. The label “Positive” represents the mutagenic category, while “Negative” represents the nonmutagenic category.

### 4.2 Comparison of Model Performances with/without Feature Selection

Herein, a comparison analysis of models with and without feature selection was presented. The feature selection was processed by first removing the constant values. Next, the features with their absolute Pearson correlation coefficients greater than 0.9, which were the dark red features in the correlation matrix (Figure S2), were filtered by only keeping the one with the largest variance. Eventually, 140 features were retained for MACCS, and 346 features were retained for Mordred. The remaining features in feature selection were utilized to establish the model. The results showed that the models without feature selection had similar performances to those with feature selection (Table S6), where the differences in testing accuracy ranged from 0.002 to 0.015. This indicated that the highly correlated features, including the most correlated pair, contributed minimally to noisy prediction in our models. The results were aligned to a study that reported deep learning models trained without feature selection have been shown to maintain better predictive performance, which may be due to feature selection inadvertently discarding valuable information.^50^

In addition, to observe whether the performances of established models were based on the underlying relationship between the features and mutagenicity, a chance correlation test was presented. The evaluation applied X-randomization to the MACCS and Mordred features, where we randomly shuffled the features for all compounds while their mutagenicity labels were kept unchanged. The results showed that models with X-randomization had accuracy, balanced accuracy, and precision values ranging from 0.500 to 0.568. In comparison, our MACCS and Mordred models had accuracy, balanced accuracy, and precision values of 0.850 to 0.944, which were substantially higher than those of the randomly shuffled models (Table S7). The findings suggest that the established models predict the compound mutagenicity based on underlying relationships as opposed to chance correlation, and that they exhibit reliable performance.

### 4.3 Model Misprediction Insights through Activity Cliffs Identification

To examine the activity cliffs in our data set, we utilized the MACCS and Mordred descriptors and the ARKA approach^51^ to generate the ARKA plot. In an ARKA plot, mutagens are expected to be in the range surrounded by the ARKA_1 descriptor greater than 0.5 and the ARKA_2 descriptor lower than −0.5, while nonmutagens are expected to be in the area circled by the ARKA_1 descriptor lower than −0.5 and the ARKA_2 descriptor greater than 0.5. In these regions, when mutagens/nonmutagens appear in a different location, they will be considered as potential activity cliffs. In Figure S3A,B, most of the mutagens appeared in the first, the third, and the fourth quadrant, while the nonmutagens had a larger proportion within the second and the third quadrant. In addition, the number of activity cliffs was summarized for each ARKA plot. In the ARKA plots using the MACCS features, the training data set contained 13 activity cliffs, while the testing data set had four. For the Mordred descriptors, the training data set also had 13 activity cliffs, but none were found in the testing data set.

Next, we showed three examples of activity cliffs in Figure S3C. 2-(dimethylamino)­ethyl methacrylate, a mutagen, was identified as an activity cliff and falsely predicted as a nonmutagen by the MACCS, Mordred, and the integrated model. The phenomenon could be explained compared to its closest compound based on the sum of absolute differences in ARKA descriptor, the Ethylenemisoctadecanamide, which is a nonmutagen and thus potentially affected the prediction. Similarly, Di­(*n*-octyl)­tin-*s*,*s*′-bis­(isooctylmercaptoacetate) and Tris­(3,4-dibromo-2-butyl)­phosphate are also mutagenic compounds but were eventually predicted as nonmutagenic. These findings indicated that the analysis of activity cliffs could provide insights into the mispredictions of our model.

### 4.4 Comparison to Other Models in Previous Studies

Herein, a comparison between our established model and the other models reported in three previous studies was conducted.^1,4,49^ The comparison was analyzed by collecting their available model performances (*i.e.*, the accuracy, balanced accuracy, F1, and MCC) in the testing data set. Generally, our MACCS-Mordred model performed a better balanced accuracy and MCC than the other reported models (Table S8). In addition, similar to Kumar et al.,^4^ the ratio between the number of mutagens and nonmutagens in our study is close to 1.3:1, unlike the other two studies with extremely unbalanced data sets, which caused their performances to be unstable. These results indicate that our MACCS-Mordred model is more effective than the previously reported models.

### 4.5 Relationship between Feature Importance and the Inducing Mutagenic Mechanism

Understanding the importance of various chemical structures can help identify mutagenic compounds. The analysis of feature importance suggested that features with positive contributions could be divided into four categories: halogen group, nitrogen-containing group, oxygen-containing group, and ring-related group. Some compounds containing these groups were demonstrated to induce mutagenicity. For example, the nitro group, aliphatic halogen, aromatic nitrogen, and heterocycle were shown to possess this capability.^52–54^ The mutagenic capability of the nitro group is attributed to its strong electrophilicity, which generates localized electron-deficient sites inside the molecule through electron-transferring interactions, subsequently causing damage to nucleic acids.^55^ In addition, compounds with aliphatic halogen groups were also observed to cause mutagenicity. For instance, the mutagenicity of vinyl chloride and vinylidene chloride was attributed to the formation of unsymmetric and highly electrophilic oxiranes, which are substances that can cause genotoxic effects by reacting directly with the nucleophilic constituents.^56,57^ Furthermore, the mutagenic potentials of aromatic nitrogen and heterocycle may result from the ability to generate redox cycling, which disrupts oxidative stress and damages the DNA. Last, the toxicity of oxygen-containing substances (*e.g.*, O-PAHs) is related to the capability of generating reactive oxygen and inducing an excessive amount of oxidative stress.^58^ The important features identified in this study match with known mutagenic-inducing mechanisms.

### 4.6 The Application of Feature Importance

The important features identified by this study highlight how changes in functional groups influence mutagenicity. We utilized the Exmol package to design compounds with modifications that would change the predicted class of a molecule. For instance, acetaminophen and anthranilic acid are nonmutagenic and can become mutagenic through specific modification (Figure S4), such as substituting an OH group with fluoride (FP-134) or adding halogen and nitrogen-containing groups (FP-84). Similarly, for the mutagenic compound Isoniazid, replacing the amine group with sulfide (FP-59) or adding methyl group (FP-149) could reduce its mutagenicity, which aligns with our findings on the negative contributors. The modifications were derived from a virtual local chemical subspace, where the compounds within it are generated based on the discussed chemical, not guaranteed for stability and synthetic feasibility, and are not included in our data set. Therefore, these virtual analogs serve only as theoretical examples to illustrate how feature importance can guide hypothesis generation, rather than definitive predictions. This analysis of molecular analogs highlights how minor modifications can significantly affect predicted mutagenicity, enhancing our understanding of the mutagenic potential of various compounds.

### 4.7 The Potential of Mutagenic Compounds for Environmental Contamination

The feature importance analysis not only highlighted the mutagenic potential of certain chemical structures but also their environmental impact. For example, several nitropolycyclic aromatic hydrocarbons (N-PAHs), which belong to the nitrogen-containing and ring-related groups, were listed as priority pollutants by the US Environmental Protection Agency (EPA). The increased complexity attributed to the aromatic group hinders degradation, while the nitro group enhances adsorption affinity on particulate matters or soil due to a reduced octanol–water partitioning coefficient and Henry law coefficient.^59^ Properties of contaminants contribute to their persistence and ubiquity in the environment and elevate their mutagenic potential. Our results suggested that the feature importance analysis could be further utilized to assess environmental contaminants and evaluate their associated risks.

## 5 Conclusions

In this study, we established an integrated machine learning model to predict the mutagenicity of organic compounds, aiming to reduce the time and cost associated with traditional assays such as the Ames test. The integrated model, combining predictions based on MACCS fingerprints and Mordred descriptors, achieved strong predictive performance, with an accuracy of 0.882, a balanced accuracy of 0.885, a precision of 0.922, a recall of 0.862, and an F1-score of 0.895. The mispredictions of our integrated model were in part attributable to activity cliffs. Our feature analysis revealed that compounds containing structural elements such as aromatic rings, nitro groups, or aliphatic halogens tend to exhibit mutagenic properties—structures commonly found in known or suspected environmental contaminants. Additionally, applicability domain analysis confirmed that reliable predictions were obtained for compounds below the critical hat value of each feature, which was 0.095 for MACCS descriptors and 0.6 for Mordred descriptors. Taken together, these findings suggest that our model provides a reliable and interpretable tool for mutagenicity assessment and may contribute to the early identification of potentially hazardous compounds in environmental monitoring and regulatory screening efforts.

## Supplementary Material



## Data Availability

The data set supporting the conclusions of this article is available in the GitHub repository: https://github.com/CHAOHSUTW/Mutagenicity_Intergrated-Model.git.
