# Supplementary material for: Establishment of an Integrated Model for Predicting Compound Mutagenicity with a Feature Importance Analysis
Source: J Chem Inf Model. 2025 Oct 21;65(21):11558–71. doi: 10.1021/acs.jcim.5c01586 (PMC12606639; doi:10.1021/acs.jcim.5c01586)
Supplement: Supplementary file 1 [file ci5c01586_si_001.pdf]

Supplementary Information

Establishment of an Integrated Model for Predicting Compound Mutagenicity with a Feature Importance Analysis

Chao-Hsu Yang[1], Tony Eight Lin [2,3], Jui-Hua Hsieh [4], Kai-Cheng Hsu [2,3,5,6,*], Pei-Te Chiueh [1,*]

[1]Graduate Institute of Environmental Engineering, College of Engineering, National Taiwan University, 71, Chou-Shan Road, Da'an Dist., Taipei 106, Taiwan

[2]Graduate Institute of Cancer Biology and Drug Discovery, College of Medical Science and Technology, Taipei Medical University, No.250, Wuxing St., Xinyi Dist., Taipei 110, Taiwan

[3]Ph.D. Program for Cancer Molecular Biology and Drug Discovery, College of Medical Science and Technology, Taipei Medical University, No.250, Wuxing St., Xinyi Dist., Taipei 110, Taiwan

[4]Division of Translational Toxicology, National Institute of Environmental Health Sciences, National Institutes of Health, 111 TW Alexander Drive, Durham, NC 27709, USA

[5]TMU Research Center of Cancer Translational Medicine, Taipei Medical University, No.250, Wuxing St., Xinyi Dist., Taipei 110, Taiwan

[6]Cancer Center, Wan Fang Hospital, Taipei Medical University, No.111, Sec. 3, Xinglong Rd., Wenshan Dist., Taipei City 116, Taiwan

*Corresponding author.

Email: piki@tmu.edu.tw (Kai-Cheng Hsu)

Email: ptchueh@ntu.edu.tw (Pei-Te Chiueh)

Supplementary Information

A

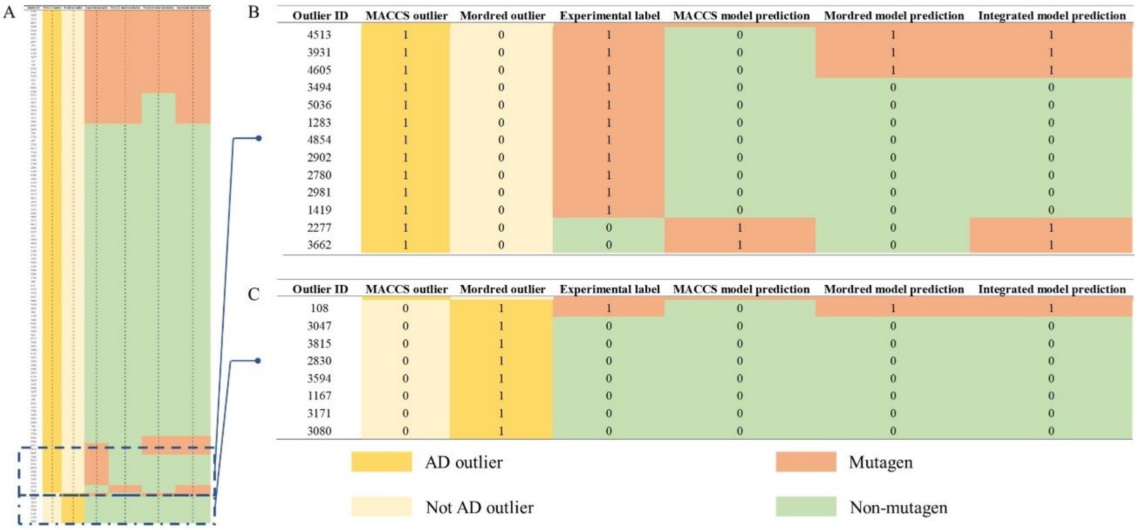

B

Outlier ID	MACCS outlier	Mordred outlier	Experimental label	MACCS model prediction	Mordred model prediction	Integrated model prediction
4513	1	0	1	0	1	1
3931	1	0	1	0	1	1
4605	1	0	1	0	1	1
3494	1	0	1	0	0	0
5036	1	0	1	0	0	0
1283	1	0	1	0	0	0
4854	1	0	1	0	0	0
2902	1	0	1	0	0	0
2780	1	0	1	0	0	0
2981	1	0	1	0	0	0
1419	1	0	1	0	0	0
2277	1	0	0	1	0	1
3662	1	0	0	1	0	1

C

Outlier ID	MACCS outlier	Mordred outlier	Experimental label	MACCS model prediction	Mordred model prediction	Integrated model prediction
108	0	1	1	0	1	1
3047	0	1	0	0	0	0
3815	0	1	0	0	0	0
2830	0	1	0	0	0	0
3594	0	1	0	0	0	0
1167	0	1	0	0	0	0
3171	0	1	0	0	0	0
3080	0	1	0	0	0	0

AD outlier

Mutagen

Not AD outlier

Non-mutagen

Figure S1. **Heatmap analysis of the applicability domain outliers.** (A) Compounds highlighted in yellow represent the outliers of the applicability domain defined by the MACCS/Mordred features, while those colored in light-yellow were within the applicability domain. (B-C) Compounds highlighted in red show they were experimentally labeled or predicted as mutagens, while those in green demonstrate they were experimentally labeled or predicted as non-mutagens

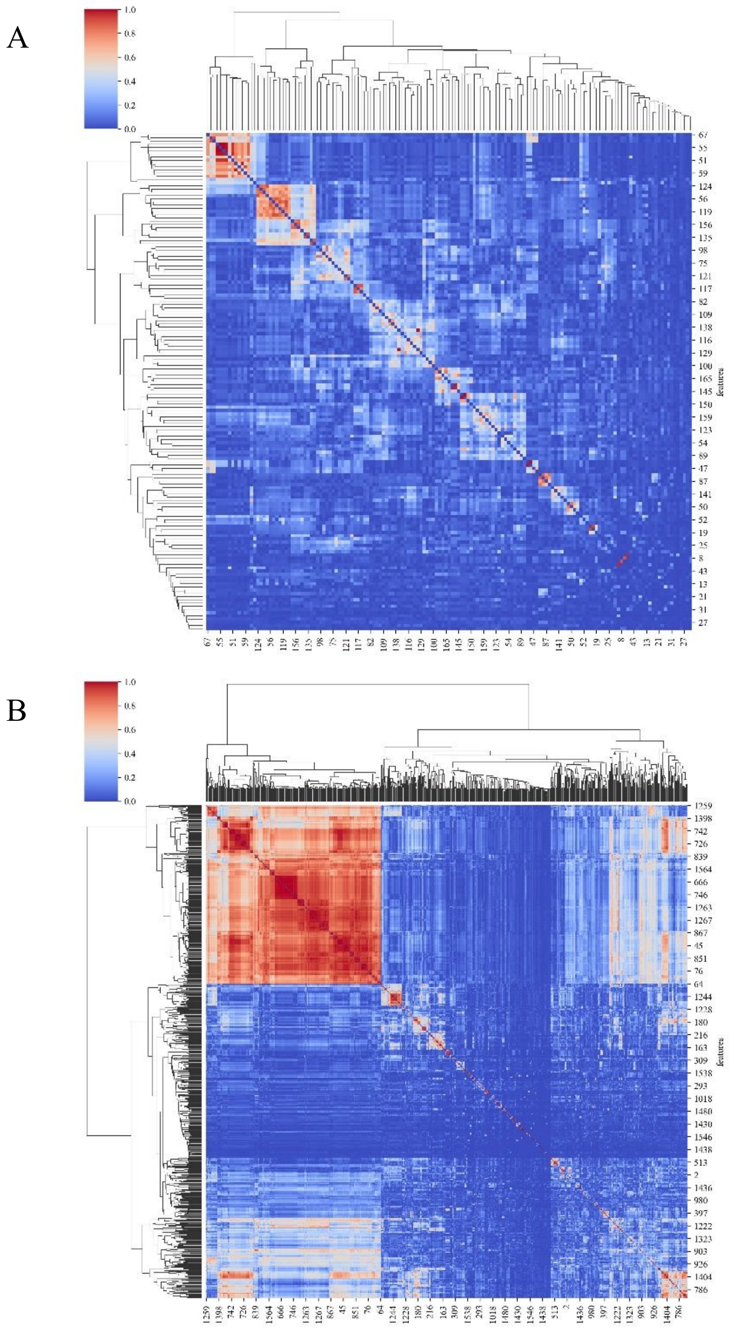

Figure S2. **Pearson correlation matrices of MACCS and Mordred features.** (A) MACCS and (B) Mordred descriptors. Features exhibiting correlations above 0.6 are shaded from light red to dark red, whereas those below 0.4 are shaded from light blue to dark blue.

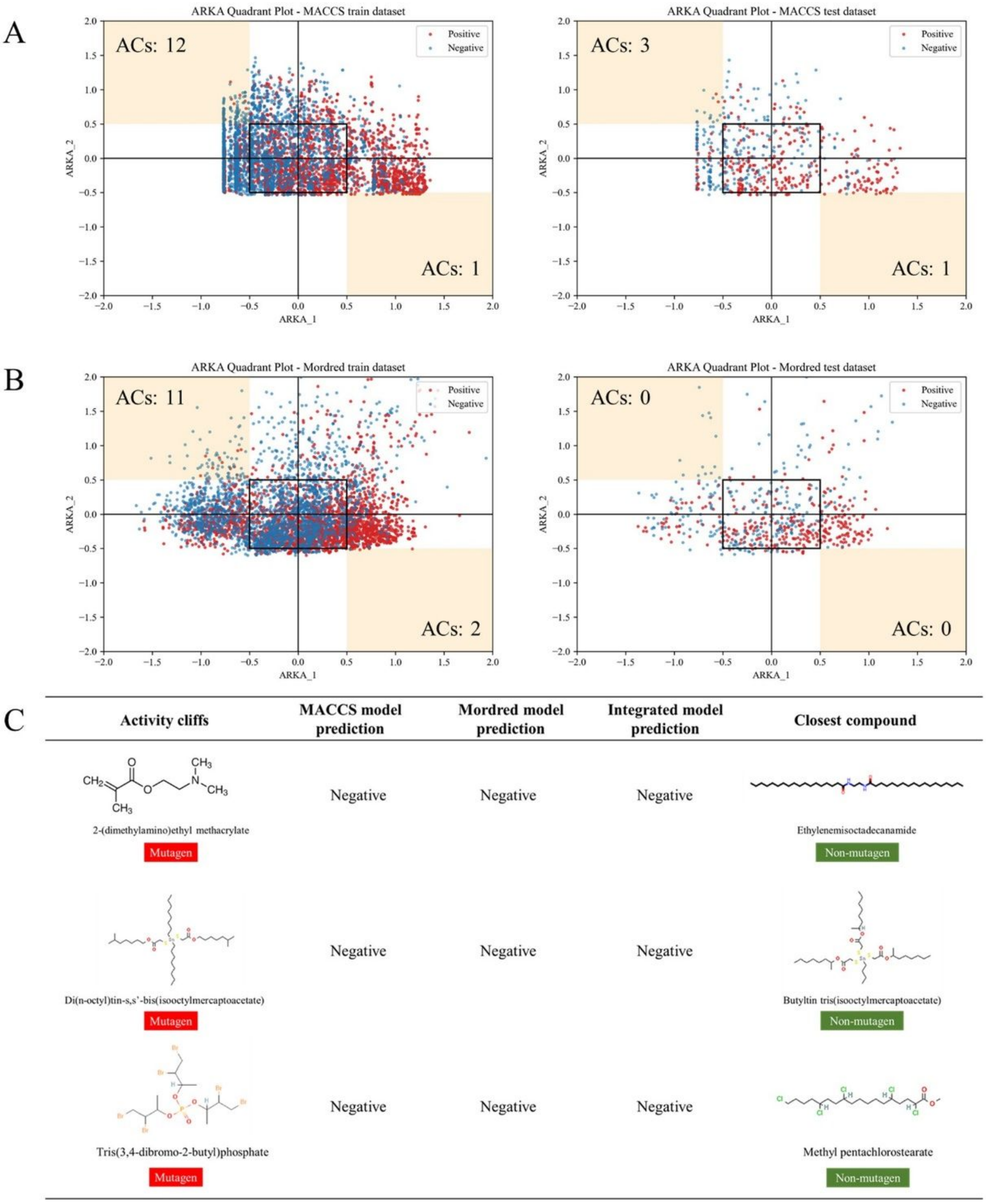

Figure S3. **Activity cliffs analysis of MACCS and Mordred model.** ARKA plots of (A) MACCS model and (B) Mordred model. The plots display the chemical distribution. The yellow-colored regions represent the area where chemicals will be identified as activity cliffs. The number of activity cliffs is summarized. (C) Three identified activity cliffs with their prediction in MACCS, Mordred and the integrated model. Their closest compounds in the ARKA plot were also provided.

Compound	Structure	Modification 1	Modification 2	Modification 3
**Acetaminophen** (Non-mutagenic)	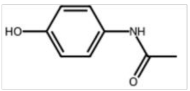	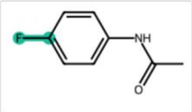 (Substitution of fluoride)	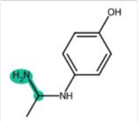 (Substitution of nitrogen)	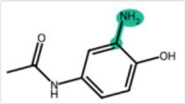 (Addition of nitrogen)
**Anthranilic acid** (Non-mutagenic)	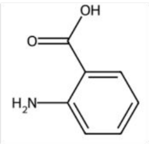	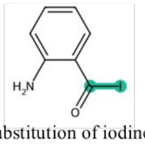 (Substitution of iodine)	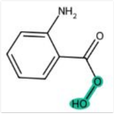 (Addition of hydroxyl)	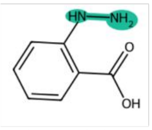 (Addition of nitrogen)
**Isoniazid** (Mutagenic)	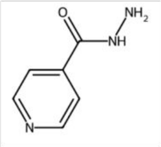	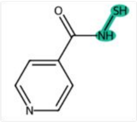 (Substitution of sulfide)	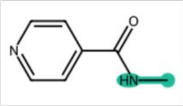 (Substitution of CH_3)	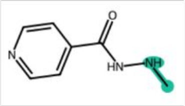 (Addition of CH_3)

Figure S4. **Structural modification analysis of compound mutagenicity.** Modifications highlighted in green represent the additions and substitutions of specific structures which alter the mutagenicity of the compounds. In contrast, structures highlighted in red show that their removals affect the mutagenicity.

Table S1. Molecular features used for model establishment.

Descriptor type	Number of used features
MACCS	167
Avalon	512
FCFP	2,048
ECFP	2,048
Topological	2,048
Atompair	2,048
RDkit	2,048
Pattern	2,048
Layered	2,048
Mordred	1,054
Roberta-Zinc480M-102M	768
GPT2-Zinc480M-87M	768
MOLT5	1,024

Table S2. Cross-validation results of the top 10 integrated models.

Integrated models	CV accuracy	CV balanced accuracy	CV precision	Average
MACCS + Mordred	0.850	0.848	0.872	0.857
MACCS + Pattern	0.852	0.849	0.866	0.856
Pattern + Mordred	0.850	0.848	0.868	0.855
MACCS + Layered	0.855	0.849	0.860	0.855
MACCS + Avalon	0.855	0.849	0.857	0.854
Layered + Mordred	0.851	0.847	0.862	0.853
MACCS + MOLT5	0.849	0.846	0.864	0.853
Pattern + MOLT5	0.850	0.846	0.862	0.853
Pattern + Layered	0.851	0.846	0.858	0.852

Table S3. Performances of single-feature models. The “Acc” represents the accuracy, “Bal acc” represents the balanced accuracy, “Pre” represents the precision, and “Rec” represents the recall.

	Training set						Testing set					
	**Acc**	**Bal acc**	**Pre**	**Rec**	**F1**	**MCC**	**Acc**	**Bal acc**	**Pre**	**Rec**	**F1**	**MCC**
Avalon	0.955	0.958	0.983	0.938	0.960	0.911	0.836	0.838	0.876	0.823	0.849	0.673
FCFP	0.976	0.977	0.985	0.973	0.979	0.952	0.799	0.801	0.847	0.780	0.812	0.599
ECFP	0.968	0.970	0.988	0.955	0.971	0.936	0.814	0.818	0.868	0.786	0.825	0.632
Topological	0.965	0.968	0.987	0.951	0.969	0.931	0.830	0.834	0.887	0.795	0.839	0.664
Atompair	0.971	0.972	0.985	0.963	0.974	0.941	0.825	0.825	0.857	0.823	0.839	0.647
RDkit	0.941	0.946	0.986	0.909	0.946	0.884	0.840	0.844	0.895	0.807	0.849	0.684
Pattern	0.935	0.941	0.989	0.896	0.940	0.875	0.835	0.839	0.889	0.804	0.844	0.673
Layered	0.952	0.956	0.983	0.932	0.957	0.906	0.848	0.851	0.889	0.832	0.859	0.697
Roberta-Zinc480M-102M	0.933	0.939	0.983	0.898	0.938	0.870	0.814	0.820	0.878	0.774	0.823	0.635
GPT2-Zinc480M-87M	0.924	0.931	0.987	0.878	0.929	0.855	0.784	0.790	0.857	0.734	0.791	0.577
MOLT5	0.91	0.918	0.980	0.859	0.915	0.828	0.804	0.811	0.879	0.752	0.811	0.618

Table S4. **The applicability domain of each model**

Descriptor type	Critical hat value (h*)
MACCS	0.095
Avalon	0.292
FCFP	1.165
ECFP	1.165
Topological	1.165
Atompair	1.165
RDkit	1.165
Pattern	1.165
Layered	1.165
Mordred	0.600
Roberta-Zinc480M-102M	0.437
GPT2-Zinc480M-87M	0.437
MOLT5	0.583

Table S5. Information on chemicals beyond our dataset. Experimental mutagenicity, integrated model prediction, and compound inclusion in MACCS and Mordred models.

**Chemicals**	**Mutagenicity**	**Integrated model prediction**	**MACCS AD**	**Mordred AD**
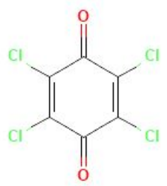 Chloranil	Positive	Positive	In AD	In AD
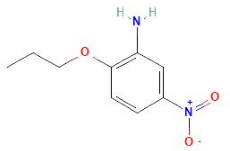 5-nitro-2-propoxyaniline	Positive	Positive	In AD	In AD
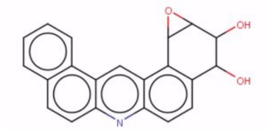 Syn-dibenz[a,j]acridine-3,4-diol-1,2-epoxide	Positive	Positive	In AD	In AD

Table S6. Comparison of model performances with and without feature selection.

Models without feature selection	train acc[1]	train balacc	train pre	train rec	train f1	train mcc
MACCS	0.911	0.918	0.974	0.866	0.917	0.828
Avalon	0.955	0.958	0.983	0.938	0.960	0.911
ECFP	0.976	0.977	0.985	0.973	0.979	0.952
FCFP	0.968	0.970	0.988	0.955	0.971	0.936
Topological	0.965	0.968	0.987	0.951	0.969	0.931
Atompair	0.971	0.972	0.985	0.963	0.974	0.941
RDkit	0.941	0.946	0.986	0.909	0.946	0.884
Pattern	0.935	0.941	0.989	0.896	0.940	0.875
Layered	0.952	0.956	0.983	0.932	0.957	0.906
Mordred	0.896	0.905	0.976	0.837	0.902	0.804
Roberta-Zinc480M-102M	0.933	0.939	0.983	0.898	0.938	0.870
GPT2-Zinc480M-87M	0.924	0.931	0.987	0.878	0.929	0.855
MOLT5	0.910	0.918	0.980	0.859	0.915	0.828

[1]The “acc” represents the accuracy, “balacc” represents the balanced accuracy, “pre” represents the precision, and “rec” represents the recall.

Table S6. continued.

Models with feature selection	train acc[1]	train balacc	train pre	train rec	train f1	train mcc
MACCS	0.911	0.918	0.971	0.870	0.918	0.828
Avalon	0.954	0.958	0.986	0.933	0.959	0.910
ECFP	0.974	0.975	0.984	0.971	0.977	0.948
FCFP	0.969	0.970	0.985	0.960	0.972	0.937
Topological	0.964	0.966	0.986	0.951	0.968	0.928
Atompair	0.975	0.976	0.985	0.970	0.978	0.949
RDkit	0.954	0.957	0.984	0.934	0.958	0.908
Pattern	0.950	0.954	0.988	0.923	0.954	0.902
Layered	0.954	0.957	0.983	0.935	0.958	0.908
Mordred	0.928	0.933	0.979	0.892	0.933	0.859
Roberta-Zinc480M-102M	0.940	0.945	0.986	0.908	0.945	0.883
GPT2-Zinc480M-87M	0.926	0.933	0.987	0.881	0.931	0.858
MOLT5	0.912	0.919	0.977	0.865	0.918	0.831

Table S6. continued.

Models without feature selection	test acc[1]	test balacc	test pre	test rec	test f1	test mcc
MACCS	0.872	0.879	0.944	0.820	0.877	0.753
Avalon	0.836	0.838	0.876	0.823	0.849	0.673
ECFP	0.799	0.801	0.847	0.780	0.812	0.599
FCFP	0.814	0.818	0.868	0.786	0.825	0.632
Topological	0.830	0.834	0.887	0.795	0.839	0.664
Atompair	0.825	0.825	0.857	0.823	0.839	0.647
RDkit	0.840	0.844	0.895	0.807	0.849	0.684
Pattern	0.835	0.839	0.889	0.804	0.844	0.673
Layered	0.848	0.851	0.889	0.832	0.859	0.697
Mordred	0.850	0.860	0.944	0.777	0.852	0.717
Roberta-Zinc480M-102M	0.814	0.820	0.878	0.774	0.823	0.635
GPT2-Zinc480M-87M	0.784	0.790	0.857	0.734	0.791	0.577
MOLT5	0.804	0.811	0.879	0.752	0.811	0.618

Table S6. continued.

Models with feature selection	test acc[1]	test balacc	test pre	test rec	test f1	test mcc
MACCS	0.859	0.864	0.921	0.817	0.865	0.723
Avalon	0.840	0.842	0.879	0.826	0.852	0.680
ECFP	0.811	0.813	0.855	0.795	0.824	0.622
FCFP	0.814	0.818	0.866	0.789	0.826	0.631
Topological	0.816	0.819	0.864	0.795	0.828	0.633
Atompair	0.825	0.822	0.844	0.841	0.842	0.645
RDkit	0.842	0.844	0.885	0.823	0.853	0.684
Pattern	0.836	0.840	0.886	0.810	0.847	0.675
Layered	0.847	0.848	0.883	0.835	0.858	0.693
Mordred	0.855	0.861	0.923	0.807	0.861	0.718
Roberta-Zinc480M-102M	0.819	0.824	0.877	0.786	0.829	0.643
GPT2-Zinc480M-87M	0.787	0.794	0.863	0.734	0.793	0.585
MOLT5	0.809	0.815	0.875	0.768	0.818	0.625

Table S7. Comparison of model performances with and without X-randomization.

Models	test acc[1]	test balacc	test pre	test rec	test F1	test MCC
MACCS	0.872	0.879	0.944	0.820	0.877	0.753
Mordred	0.850	0.860	0.944	0.777	0.852	0.717
MACCS-X-randomization	0.557	0.500	0.557	1.000	0.716	0.000
Mordred-X-randomization	0.557	0.500	0.557	1.000	0.716	0.000

[1]The "acc" represents the accuracy, "balacc" represents the balanced accuracy, "pre" represents the precision, and "rec" represents the recall.

Table S8. Comparison of our MACCS-Mordred model with other reported models

Models	Number of mutagens/non-mutagens	Testing accuracy	Testing balanced accuracy	Testing F1	Testing MCC
MACCS-Mordred model	3,323:2,543	0.882	0.885	0.891	0.766
Kumar et al (2021)	2,293:1,760	0.838	NaN	0.811	NaN
Martinez et al (2022)	3,103:231	0.950	0.710	0.980	0.600
Li et al (2023)	1,480:8,546	0.840	0.690	0.480	0.380
